# Tuberculosis in the pituitary fossa: a common pathology in an uncommon site

**DOI:** 10.1530/EDM-14-0091

**Published:** 2014-12-01

**Authors:** K Majumdar, M Barnard, S Ramachandra, M Berovic, M Powell

**Affiliations:** 1 University College London Hospitals NHS Foundation Trust, Diabetes and Endocrinology, 250 Euston Road, London NW1 2PG, UK; 2 Whittington Hospital, London, UK; 3 National Hospital for Neurology and Neurosurgery, London, UK

## Abstract

Tuberculosis (TB) is an important cause of mortality and morbidity across the world. In 2–5% of all cases of systemic TB, the C is affected, with lesions reported in the meninges, cortex and ventricles. Intrasellar tuberculomas, however, are extremely rare. We report the interesting case of a young female patient who presented with secondary hypothyroidism and hyperprolactinaemia. She was treated successfully for pituitary TB. We also highlight and discuss some interesting (and hitherto unreported) endocrine issues. Radiological and histological features and treatment of pituitary TB are discussed using this case as an example.

**Learning points:**

- Intrasellar TB continues to be a rare presentation, but incidence and prevalence are expected to grow with increasing numbers of multidrug-resistant TB and shrinking geographical boundaries across the world.
- Pituitary TB can present with features of a typical adenoma, but has certain radiological and histological features that help to differentiate from an adenoma.
- Patients can present with a variety of endocrine abnormalities at different times.
- The presence of an intrasellar mass in individuals at a high risk of developing TB, or with a previous history of systemic TB, should prompt the diagnosis of pituitary TB. In such individuals, it may be worth considering a trial of anti-tuberculous therapy, before considering surgery.

## Background

Our case highlights a rare presentation of one of the commonest multi-system infections in the world. In 2011, an estimated 8.7 million new cases of tuberculosis (TB) were reported (1). The incidence of co-infection with the human immunodeficiency virus and multi-drug resistant TB is also increasing (1). The last few decades have seen significant levels of migration in the world. Therefore, diseases that were once common only in the developing countries are also manifesting themselves (along with their variations) in the developed countries. Previous inexperience in dealing with these cases often poses diagnostic and therapeutic challenges.

We present a complex case of a young woman who developed headaches and amenorrhoea on a background of presumed pulmonary TB. She acquired multiple-hormone deficiencies through the course of diagnosis and treatment of a pituitary lesion, which was eventually postulated to be a pituitary tuberculoma. She had radiological features in keeping with pituitary TB, but took longer to respond to treatment.

## Case presentation

A 25-year-old African woman was referred to the Endocrine Clinic with a 4-month history of bi-temporal headaches and amenorrhoea. The headaches were episodic and associated with photophobia and nausea. She attained menarche at the age of 11 and her periods had previously been regular. She had been trying to conceive for 2 years without success. She also had a weight gain of nearly 9 kg over a span of 6 months. Clinical examination revealed no systemic abnormalities. Visual fields were full to confrontation testing.

Her past medical history was complex. A year before the endocrine review, she was reviewed in the respiratory clinic with a dry cough. Serial chest radiographs confirmed a bulky right hilum and paratracheal region with presumed enlarging lymphadenopathy. There were no systemic symptoms of weight loss, night sweats or fevers. She had been in the UK for 5 years before presentation, and had had no contacts with TB. She complained of a painful right shoulder, and magnetic resonance (MR) scan of the affected side revealed a soft tissue mass in the suprascapular bursa which extended to the inferior glenoid pouch. Ultrasound-guided biopsy was undertaken and revealed a core of normal tissue with no evidence of malignancy or infection. No acid-fast bacilli (AFB) were seen. She declined to undergo mediastinoscopy and was treated empirically with anti-tuberculous therapy (ATT) for a total of 9 months (initially 3 months and subsequently completed a 6 month course). Significant radiological improvement of chest lymphadenopathy was noted on serial CT imaging.

## Investigation

In the Endocrine Clinic, she was noted to have secondary hypothyroidism, hypogonadotrophic hypogonadism and mild hyperprolactinaemia (see Table 1). Response to the short Synacthen test was normal. MR scan of the pituitary fossa revealed a large sellar mass, extending into the suprasellar cistern, measuring 17×13.5×20 mm. The mass shows intermediate signal on T1 weighted images and enhanced avidly following contrast. The optic chiasm was noted to be draped over the superior aspect of the mass. There was no focal cerebral lesion or meningeal enhancement following contrast.

**Table 1:** Endocrine investigations at first presentation (normal ranges in brackets)

**Hormone**	**Value**	**Normal ranges**
Free tri-iodothyronine (pmol/l)	2.8	3.1–6.8
Free thyroxine (pmol/l)	5.5	12–22
Thyroid-stimulating hormone (TSH) (mU/l)	3.7	0.3–4.2
Prolactin (mU/l)	504	102–496
Random cortisol (nmol/l)	310	
Insulin-like growth factor 1 (IGF1) (nmol/l)	12.9	
Luteinising hormone (LH) (IU/l)	4.8	
Follicle-stimulating hormone (FSH) (IU/l)	7	

## Treatment

The patient underwent a trans-sphenoidal hypophysectomy at our regional neuro-endocrine centre. Interestingly, intra-operatively, our experienced pituitary surgeon commented on the hardened nature of pituitary tissue, as a result of which a substantial portion could not be removed. This was the first time in the course of events so far, that there was a doubt cast on the possibility of the lesion being a typical adenoma. Following surgery, her headaches improved significantly, but she remained amenorrhoeic. Biochemical tests post-operatively demonstrated a low cortisol level (<200 nmol/l), and she was commenced on glucocorticoids. Subsequently an insulin tolerance test was performed, which demonstrated a normal pituitary–adrenal axis (peak cortisol 552 nmol/l). Glucocorticoid cover was subsequently stopped. Luteinising hormone-releasing hormone (LHRH) test was also performed in view of persisting amenorrhoea, which revealed a peak LH of 4.9 IU/l and follicle-stimulating hormone (FSH) of 5.4 IU/l. Post-operative MRI obtained 3 months after surgery continued to show a substantial pituitary lesion measuring 12×8.6×13 mm in size.

**Figure 1:**
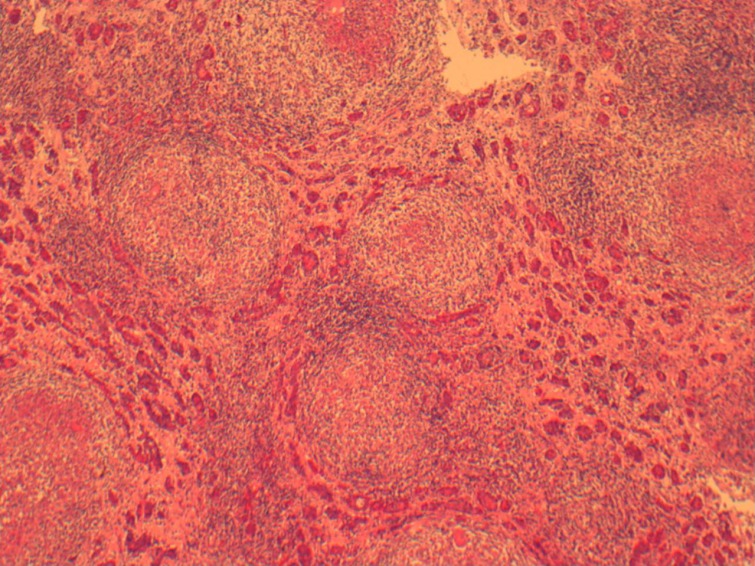
Histology of resected mass showing multiple granulomas.

**Figure 2:**
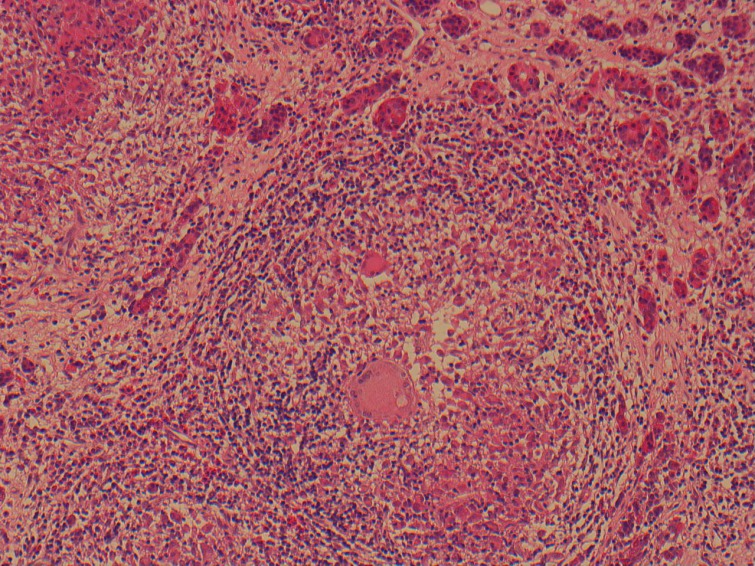
Large granuloma with area of central necrosis.

Histology of the resected tissue revealed a necrotising granulomatous hypophysitis (Figs 1 and 2). The granulomas were predominantly composed of epithelioid histiocytes and contained several Langhan's giant cells, each surrounded by a well-developed mantle of small lymphocytes (CD3+). The areas of caseous necrosis were not seen within the granulomas. No micro-organisms were identified with Ziehl Neelsen (ZN) stains, and no AFB were seen. The slides were reviewed at regional neuropathology meeting, and the overall impression was that given the history of probable systemic TB, tuberculous inflammation of the pituitary was most likely. In retrospect, we realised that the absence of AFB or lack of direct confirmation on histopathological tests was not unusual in the case of sellar TB, as is explained in the discussion later. The patient was also evaluated for immune deficiencies at a regional tertiary centre. She had an excellent response to phytohaemagglutin (PHA)-induced interferon gamma, and CD-4 cell count was 1250 cells/μl, which is well within normal limits (450–1600 cells/μl).

**Figure 3:**
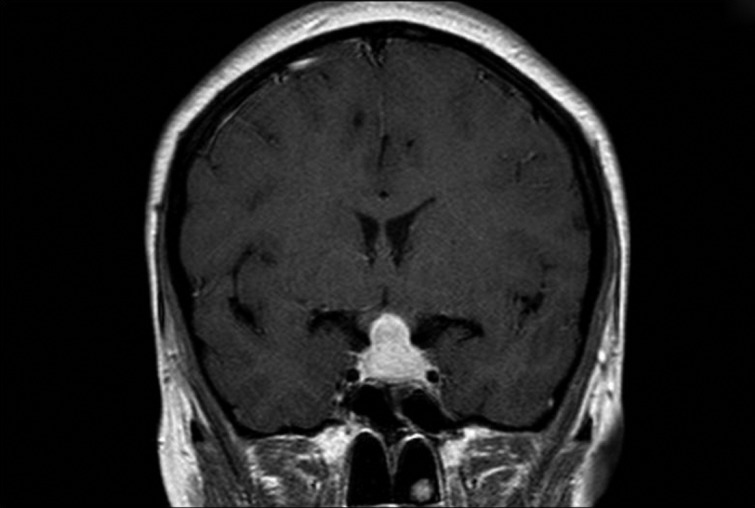
MRI scan of pituitary fossa at presentation.

**Figure 4:**
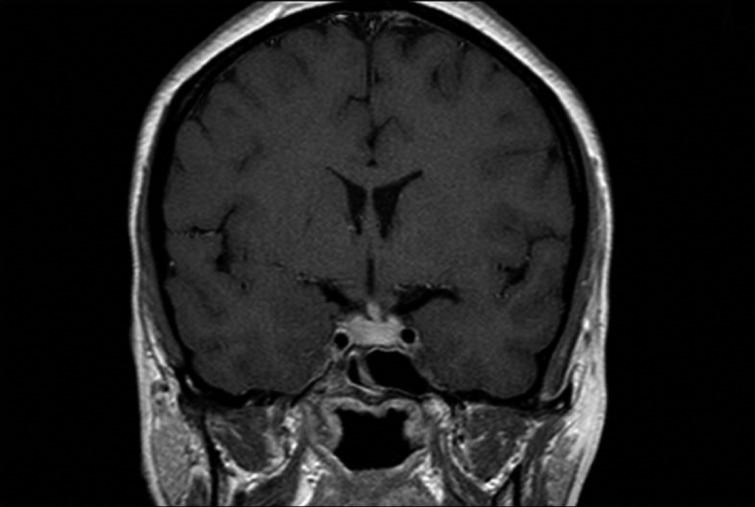
MRI scan of pituitary fossa 7 months after completing 1 year ATT.

ATT was recommenced (initially supervised directly observed treatment), with a plan to continue for a minimum period of 1 year and possibly extending to 18 months. Standard course of ATT was provided with an initial 2-month phase of intensive therapy with isoniazid, rifampicin, pyrazinamide and ethambutol, followed by isoniazid and rifampicin for the remaining duration of therapy. The patient also received additional steroid cover with prednisolone, for possible sarcoidosis. However, the patient stopped ATT at the end of 1 year. She was extremely keen to get pregnant and ATT was delaying her plans. We monitored her response to therapy by serial MR scanning of her pituitary fossa, and noted a significant but delayed improvement in the size of the residual tumour mass at 8 months after completion of ATT (Figs 3 and 4).

## Outcome and follow-up

Surprisingly, the patient remained amenorrhoeic throughout the course of her investigations and subsequent treatment. She was eventually referred to the local fertility clinic where she received ovulation induction and delivered a healthy baby boy a year later. During pregnancy, she was again noted to be cortisol deficient (random serum cortisol 98 nmol/l) and received oral glucocorticoid replacement with hydrocortisone. (She was admitted to hospital with an episode of vomiting, and admits to being non-compliant with her medications) She was admitted to hospital with an episode of vomiting, and confessed to being non-compliant with her medications. The transient cortisol deficiency noted during pregnancy resolved post-partum. She did not breast-feed. She currently remains under endocrine follow-up and is replaced with thyroid hormone.

## Discussion

Intracranial tuberculomas account for 0.15–4% of all intracranial tumours (2) (3). TB affecting the sellar region, however, is extremely rare, with no reported incidence or prevalence. Although the first reported case of a sellar tuberculoma was from Britain in 1924 (4), the literature on sellar tuberculomas, unsurprisingly, largely arises from the Indian subcontinent and other developing countries.

Our patient presented with a history of headaches, amenorrhoea and infertility and the background of recent treatment for presumed pulmonary TB. Initial biochemistry was compatible with mild hyperprolactinaemia and secondary hypothyroidism, with normal levels of cortisol, gonadotrophins and growth hormone. Sharma *et al*. (5), who have reported the largest series on intrasellar TB so far (18 cases), have described a 100% incidence of headaches and 38% incidence of endocrine disturbances including the amenorrhoea–galactorrhoea syndrome. A concomitant history of systemic TB is not always present. Radiologically (as in our patient), the tumour is usually sellar and suprasellar, being isointense on T1-weighted images and hyperintense on T2-weighted sequences (5). Pituitary stalk thickening is often noted on MR scans (6) (7). The clinical and radiological features of these lesions mimic a typical pituitary adenoma. However, following surgery, histological appearances characteristically reveal necrotising granulomatous inflammation with areas of caseation necrosis. AFB are rarely demonstrated within the lesions (5), which in turn serve to widen the differential diagnosis to include other granulomatous conditions such as sarcoidosis. In fact, our patient was empirically treated for neurosarcoidosis briefly with systemic glucocorticoids, but this made no difference to her clinical or radiological course.

From the endocrine point of view, our patient continued being amenorrhoeic and had evidence of hypogonadotrophic hypogonadism post-operatively, despite a normal response to LHRH. However, the LHRH test itself has a low specificity and we felt that she had, at the very least, partial hypogonadotrophic hypogonadism. It was also unusual that she manifested cortisol insufficiency transiently, through the course of her investigations and management. It was especially surprising to note low cortisol levels during pregnancy, which is a state characterised by raised cortisol levels (secondary to higher-cortisol binding globulin). This has not been reported in the literature as yet. We postulate that her depressed pituitary functions were a sequel of the possible inadequacy of initial treatment for pulmonary TB, when she received a discontinuous 9 month course of ATT.

Thus, although our patient did present with clinical, radiological and histological features characteristic of pituitary tubercular infection, she also had unusual endocrine manifestations. Given the increasing levels of population migration and the multi-ethnic nature of society, it is important to be aware of these hormonal variations as we are more likely to see and deal with unusual infections that are no longer restricted to the ‘developing’ countries.

## Patient consent

Informed consent has been obtained from the patient for publication of the submitted article and accompanying images.

## Author contribution statement

K Majumdar wrote the initial draft of the article, co-ordinated with the other authors and prepared the final manuscript. Permission to write this article was granted by the named physician, Dr M Barnard. M Barnard is the named physician for this patient. She gained formal consent for the publication of this article, and provided final comments on the script. S Ramachandra provided expert opinion on histology obtained following pituitary surgery. M Berovic provided expert opinion on all radiological investigations undertaken before and after surgery. M Powell was the neurosurgical lead in this case and gave us valuable insights on the intra-operative findings.
